# Thermal-Conductivity Apparatus for Steady-State, Comparative Measurement of Ceramic Coatings

**DOI:** 10.6028/jres.105.050

**Published:** 2000-08-01

**Authors:** A. J. Slifka

**Affiliations:** National Institute of Standards and Technology, Boulder, CO 80303

**Keywords:** guarded hot plate, infrared microscope, thermal-barrier coating, thermal conductivity, yttria-stabilized-zirconia

## Abstract

An apparatus has been developed to measure the thermal conductivity of ceramic coatings. Since the method uses an infrared microscope for temperature measurement, coatings as thin as 20 μm can, in principle, be measured using this technique. This steady-state, comparative measurement method uses the known thermal conductivity of the substrate material as the reference material for heat-flow measurement. The experimental method is validated by measuring a plasma-sprayed coating that has been previously measured using an absolute, steady-state measurement method. The new measurement method has a relative standard uncertainty of about 10 %. The measurement of the plasma-sprayed coating gives 0.58 W·m^−1^·K^−l^ which compares well with the 0.62 W·m^−1^·K^−l^ measured using the absolute method.

## 1. Introduction and Motivation

Ceramic coatings have applications primarily as wear coatings and thermal barrier coatings. Thermal-barrier coatings (TBCs), which are ceramic coatings applied to metal substrates, have applications in aerospace, gas turbine engines, and diesel engines. Frequently a TBC protects a metal substrate material from both high temperature and excessive wear or corrosion. One may ask why a monolithic ceramic is not used in these applications and in most wear applications. Monolithic ceramics have poor thermal shock resistance and low fracture toughness. The metallic substrate that a coating is applied to provides the necessary toughness to enable many applications where coatings are used. Ceramics are applied as graded, layered, or monolithic coatings. The thermal conductivities of these coatings must be known for engineering design and for design of new coating systems for future applications. As new coating systems are developed with lower thermal conductivities and higher mechanical reliabilities, thinner coatings can be used.

This work reports the development of a steady-state measurement method for determining the thermal conductivities of ceramic coatings. Thermal measurements on these coatings are almost always done with transient methods. Due to the processing methods used to apply ceramic coatings, primarily plasma-spray (PS) and electron-beam physical-vapor-deposition (EB-PVD), these coatings develop unique microstructures [1]. The unique microstructures and anisotropy of TBCs stretch the limits of the assumptions underlying the mathematics that apply to transient measurement of thermal diffusivity and steady-state measurement of thermal conductivity. Even though the laser-flash technique has been applied successfully for measuring the thermal diffusivity of ceramic coatings, it is useful to have a steady-state method for comparison. At NIST, we have been measuring steady-state thermal conductivity of monolithic ceramics and ceramic coatings using a guarded-hot-plate apparatus [2, 3]. Many of the coatings sent to us for measurement are 150 μm thick or less, and a coating of 150 μm is as thin as can be reliably measured in our guarded hot plate. Therefore, we need a steady-state measurement method for thinner coatings to complement our guarded hot plate and to complement the laser-flash technique commonly used to measure thermal properties of these coatings. The method developed here uses an infrared microscope to measure temperature differences on millimeter-sized specimens. Heat loss calculations in Sec. 3 show that the small size of the specimens allows the measurements to be made in air, rather than vacuum, which simplifies the apparatus needed. The noncontact nature of the infrared temperature measurement is an advantage, because any contact between a sensor and a small specimen would affect the temperature of the specimen.

TBCs are used to protect metals from oxidation and excessive thermal environments. The thermal conductivity of the metallic substrate is generally known, as it is usually an alloy that has been used for some time. A metallic substrate coated with a TBC can be used in a comparative, steady-state measurement of thermal conductivity with the substrate as the known comparative specimen. This scheme is similar to the ASTM E 1225-87 Standard Test Method for Thermal Conductivity of Solids by Means of the Guarded-Comparative-Longitudinal Heat Flow Technique (cut-bar technique) [4]. There are two significant differences between this standard method and the method developed here. One is that the test method developed here operates on a much smaller size scale, allowing, in principle, measurement of features on the order of 10 μm. Additionally, this method needs no guarding for heat losses, because the heat losses are negligible compared to the standard experimental uncertainty, despite the small size of the specimen used. The short distance over which the temperature measurement is made is a major factor for ensuring negligible heat losses.

The thermal conductivity of a PS TBC was measured using a guarded-hot-plate (GHP) apparatus which uses disk-shaped specimens of 69.85 mm diameter. The GHP method is an absolute, steady-state method for determining thermal conductivity. Furthermore, the guarded-hot-plate technique requires that the sample have a thermal barrier that is somewhat thick, 150 μm or more, because there is a finite and measurable thermal contact resistance between the specimen and sensor plates. As coatings become increasingly thin, resolving the difference between the thermal resistance due to the coating and the thermal resistance of the interface between the specimen and sensor plate becomes difficult. Therefore, a new technique applicable to a smaller size scale is needed. The device developed here uses a thermal microscopy system based on an infrared scanner which accurately determines temperature for thermal-conductivity measurements.

## 2. Development and Experimental Method

### 2.1 Development

As mentioned earlier, this method is a variation of the ASTM E 1225-87 methodology commonly known as the cut-bar technique. In the cut-bar technique, a specimen of unknown thermal conductivity is sandwiched between two pieces of material with known thermal conductivity using a thermal grease and a pliable metal foil to eliminate interfacial thermal contact resistance between the materials. Thermocouples placed along the lengths of the three material pieces yield information on the rate of heat flow through the two reference-material sections of known conductivity. The heat-flow rate can then be used to determine thermal conductivity of the unknown specimen using the one-dimensional Fourier conduction equation:

$$Q=-\lambda \;A\;\frac{dT}{dx},$$ (1)

where *Q* is the rate of heat flow, λ is the thermal conductivity, *A* is the cross-sectional area through which the heat flows, and d*T*/d*x* is the temperature gradient. Experimentally, d*T* is approximated by Δ*T*, the finite temperature difference, and d*x* is approximated by Δ*x*, the distance over which the temperature difference is measured.

In the new measurement method, only one section of known material is used, the substrate on which the coating is applied. A second section of known material on the other side of the coating is impractical. In addition, the technology of applying TBCs does not allow for the use of an aid for reducing thermal contact resistance between the coating and substrate. Most coating processes use a thin bond coat, typically MCrA1Y or some other alumina-former between the substrate and coating, to provide adhesion and to function as a diffusion barrier. Here M corresponds to a polyvalent metal, but is typically nickel. Because there is no cross-check for determination of heat flow using this method due to the fact that the substrate material of known conductivity is on only one side of the coating, the validity of the test methodology is demonstrated by measuring a coating that has been previously measured in a guarded hot plate [3].

For the measurement of the thermal conductivity of ceramic coatings, temperature must be determined accurately on a spatial scale of tens or hundreds of micrometers. Infrared microscopy is one way of determining temperature on the necessary size scale. However, it is not commonly used due to the high cost of a scanning system and the potential problem with emissivity. An infrared microscope detects radiation over a certain wavelength range, which is 8 μm to 12 μm in this case. In order to determine temperatures accurately, either the emissivity must be known or temperature must be calibrated for the sample of interest. Software schemes can be used to correct for emissivity, but they are generally not very good, and greater accuracy can be achieved with temperature calibration, which is the method used for this work. Infrared microscopy has the distinct advantage of being a noncontact method of temperature measurement.

The microscope itself is first calibrated using a sapphire blackbody provided by the manufacturer. Measurements are done over a range of temperatures and the results are compiled by the manufacturers proprietary software. The temperature of the blackbody is independently monitored with a calibrated thermocouple placed between the blackbody mount (brass) and a heating stage. The manufacturer supplies a set of three constants that yields a linear relation between temperature and detector output over the calibrated temperature range.

**Fig. 1:**
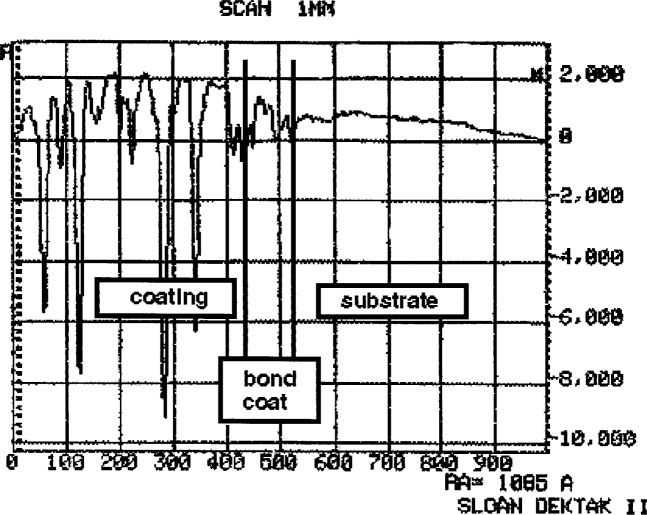
Profilometric trace of the 0.87 mm thick plasma-spray coating, after polishing but before carbon coating.

**Fig. 2:**
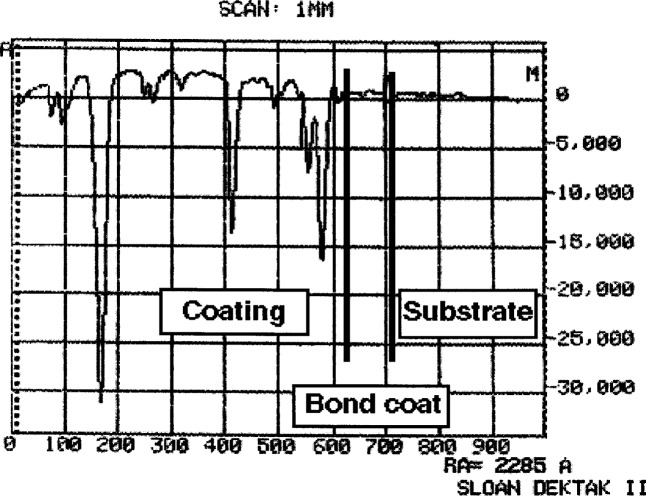
Profilometric trace of the 0.87 mm thick plasma-spray coating, after polishing and carbon coating.

To increase the emittance of both the coating and substrate surface, each specimen is cross-sectioned, polished, then carbon-coated on the side which is to be viewed by the microscope. Polishing down to a grit size of 1 μm diamond results in a surface finish with roughness of at most 0.25 μm and only a 0.1 μm difference in height between the coating and substrate. Since the substrate is significantly softer than the coating, the substrate polishes faster than the coating. Figure 1 shows a profilometry trace of a polished specimen. Subsequent carbon coating results in only a slightly rougher surface, as shown in Fig. 2.

Each carbon-coated specimen is then placed in a large isothermal block of copper or aluminum and imaged with the microscope. Small calibrated thermocouples are used to determine that the specimen is isothermal. These thermocouples are small enough to measure the temperatures of the coating and substrate, but too large to be placed with the precision needed for measurement of thermal conductivity. The emissivities of the substrate and coating phases can be determined by using the known temperature of the specimen. These known emissivities are then used to determine specimen temperatures accurately. Due to the high emissivity of the carbon coating, any differences in emissivity are small. The measurement of thermal conductivity uses temperature-difference measurements of both the coating and substrate. The temperature differences are relatively small and scale linearly with emissivity. The specimens tested generally have a measured coating emissivity of 0.97 and the substrate has an emissivity of at least 0.85 after thin carbon coating. Although a thicker carbon coating takes longer to produce, it results in a uniform emissivity of 0.97 over the entire specimen, and is preferred because it simplifies the measurement.

### 2.2 Experimental Method

**Fig. 3:**
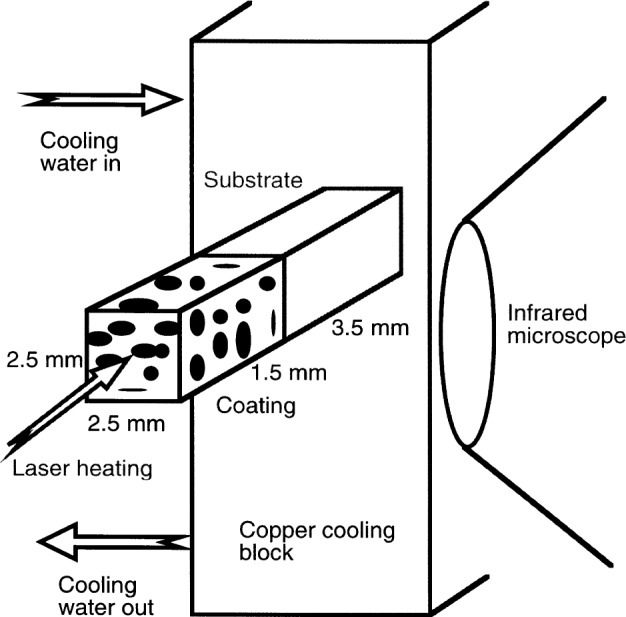
Schematic drawing of the primary features of the measurement method.

Figure 3 shows a schematic drawing of the primary features of the measurement system. The infrared microscope has a single detector that is cooled by liquid nitrogen and is sensitive to a range of wavelength from 8 μm to 12 μm. An internal rotating mirror scans the thermal image across the detector. The substrate side of the specimen is glued to a copper block through which water is pumped at constant temperature for cooling. Cyanoacrylate adhesive gives a repeatable joint between the specimen and copper cooling block and also allows specimens to be easily removed with a moderate mechanical shock. This preserves the carbon coating and allows subsequent retesting without any changes to the specimen.

The infrared microscope has a fixed field-of-view of 1.6 mm by 1.6 mm, so the specimen can be as small as 1.6 mm by 1.6 mm by the specimen thickness. The specimen could be larger and the microscope could view just the part of interest of the viewed side. In fact, since the heat that is conducted through a specimen of square cross-section and a given thickness is proportional to the length of a side squared and the heat lost to convection is proportional to the length of a side, a larger square specimen will have a higher conduction-to-convective-loss ratio. However, calculations show that heat losses are negligible for the small specimens used here, typically 3 mm by 3 mm by 5 mm. One of the reasons for a small specimen is that it is difficult to obtain large, uniform, state-of-the-art specimens from industrial and academic sources. In addition, there is a practical reason for a small specimen. The infrared microscope has a fixed operating distance of 4 mm, limiting the available space near the instrument for the sample. The positioning stage will handle a reasonable mass, but the mass of a cooling stage that can remove the necessary amount of heat from a large specimen becomes unmanageable.

The most practical method for heating the specimen is by means of a laser which provides an efficient non-contact source of heat. The desired heating power is about 0.5 W to 1 W flowing through the specimen. A 10 W, continuous-beam infrared laser is used to heat the ceramic coating. The laser beam is defocussed, or expanded, and then limited by an aperture to produce a spot that covers the entire end of the specimen. The aperture cuts out the central portion of the beam, providing uniform heating. Since an aperture is used, only about 20 % of the laser power goes into heating the specimen. The laser is computer controlled to provide repeatability between successive runs. The Invar^1^-stabilized laser provides power at less than 2 % variation over the 30 min duration of a test, which provides the stability necessary for the heat-flow to achieve steady-state. Heat flowing through the coating to the substrate is conducted away from the copper block by the cooling water.

The infrared microscope has a very short depth of field, about 10 μm. Experiments show that the measured temperatures are sensitive to focus. Measured temperature differences change by 25 % when the image is moved 30 μm out of focus. Fortunately, the specimen stage is very stiff and the focussing is fine enough to easily be within 10 μm of apparent correct focus as defined by the observer. Experiments show that changes of 10 μm from the apparent correct focus do not change the recorded temperature significantly. The infrared microscope, laser, and beamline optics are set up on an optical table to reduce vibration that would cause additional apparent thermal noise. Since water is pumped through the cooling block, vibrations cannot be completely eliminated, but the lines connecting the cooling water bath to the cooling block are long and flexible.

The infrared scanning system allows temperature sensors to be placed, via software, on the field of view in increments of 11 μm. These temperature spots are not physical sensors, but rather a software device that measures the temperature of a single pixel in the scanned field-of-view. The temperature spots can be placed at precise *x*-*y* coordinates. The software temperature sensors can be repeatably placed at fixed distances, two or more on the coating and two or more on the substrate. The consistency of the data can be checked by placing more than two temperature spots on the coating and substrate. The greater the spot spacing, the greater the temperature difference between temperature spots and, hence, the greater the precision of the measurement. The temperatures of the four spots are measured at a rate of 6 to 25 times per second for 30 min. This stream of temperature data is used to verify steady-state temperature during the test; the data are averaged to determine precise temperatures. There is significant random thermal noise internal to the infrared scanner that can be minimized by this averaging. The temperature difference Δ*T*, across a fixed, known distance Δ*x*, of the substrate is used in conjunction with the known thermal conductivity λ of the substrate material to calculate the heat flow rate *Q* through the specimen with Eq. (1). The temperature difference Δ*T*, across a fixed, known distance of the coating Δ*x* is then used along with the calculated heat flow rate *Q* and the measured cross-sectional area *A* in Eq. (1) to determine the thermal conductivity of the coating λ.

## 3. Heat Loss Calculations

A useful measurement of thermal conductivity should be as simple as possible, so the conductivity is measured in air. The specimen could be enclosed in a vacuum chamber, but the complexity of adding a vacuum system is undesirable at this point. Since the measurement is done in air and the operating equation is one-dimensional, heat-loss calculations must be performed to determine whether convection and radiation strongly affect the heat flow. For radiative heat losses, heat-flow rates are calculated based on a typical conductive heat-flow rate. Since convective heat transfer is complex, a two-dimensional analysis that gives the temperature profiles of the specimen is used. The calculations show that the heat-flow through the specimen is dominated by unidirectional solid conduction.

### 3.1 Radiative Heat Losses

The heat loss by radiation can be calculated by starting with Planck’s blackbody radiation law and determining the heat flow rate as:

$$Q=S\varepsilon \sigma \left(T^{4}-T_{\text{amb}}^{4}\right),$$ (2)

where *S* is the applicable surface area of the specimen radiating heat, *ε* is the emissivity of the specimen surface, *σ* is the Stefan-Boltzmann constant, *T* is the thermodynamic temperature of the object radiating energy, and *T*_amb_ is the thermodynamic temperature of the medium into which radiation is received. For typical operating conditions of 50 °C above room temperature, with 1 W flowing through the specimen by solid conduction, the total radiative heat loss is on the order of one milliwatt. A major factor keeping this heat loss down is the small surface area and thinness of the coating.

### 3.2 Convective Heat Losses

The specimen geometry used in these measurements has the appearance of a fin of uniform cross-section. One might think that the traditional heat-transfer analysis of a one-dimensional fin of uniform cross-section can be used in this case to determine the convective heat loss for this measurement method. It cannot, because the experimental condition used here includes a heat-generation term that does not occur in the treatment for the one-dimensional fin equation. The addition of the heat-generation term, from the heating laser, makes the heat balance different for this experimental case from that of the one-dimensional fin analysis. Therefore, a representative two-dimensional analysis is used to examine the magnitude of convective heat losses from the specimen and the resulting temperature profile.

**Fig. 4:**
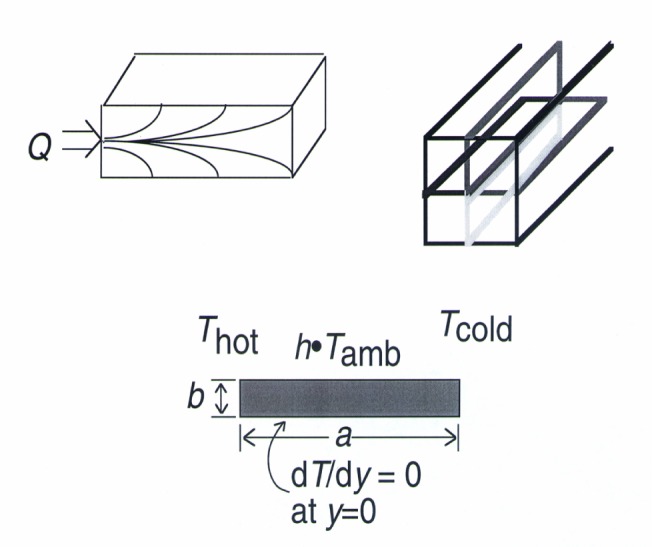
Schematic drawing of how the three-dimensional specimen is separated into representative, equivalent two-dimensional sections.

Since the specimens used for this experimental method are square in cross-section, a two-dimensional analytical approach can be used to investigate the temperature profile in the specimen and the resulting convective heat losses. Symmetry allows us to use only a quadrant of the specimen sectioned lengthwise as shown in Fig. 4. The analysis is valid for any of the four equivalent sections shown in Fig. 4. The problem is to solve the two-dimensional Laplace equation since the experiment is performed at steady-state:

$$\frac{\partial^{2}T\left(x,y\right)}{\partial x^{2}}+\frac{\partial^{2}T\left(x,y\right)}{\partial y^{2}}=0$$ (3)

subject to the boundary conditions

$$T\left(x,y\right)=T_{\text{hot}}\;\text{at}\;x=0,$$ (4)

$$T\left(x,y\right)=T_{\text{cold}}\;\text{at}\;x=a,$$ (5)

$$\frac{\partial T\left(x,y\right)}{\partial y}=0\;\text{at}\;y=0,$$ (6)

$$\text{and}\;\lambda \frac{\partial T\left(x,y\right)}{\partial y}+h\;T\left(x,y\right)=h\;T_{\text{amb}}\;\text{at}\;y=b.$$ (7)

*T*_hot_ and *T*_cold_ are the hot and cold thermodynamic temperatures of the body in question and *h* is a heat-transfer coefficient. The constants *a* and *b* are position coordinates defining the length and width of the body. The Laplace equation has many solutions. One convergent solution is

$$\begin{array}{c} T\left(x,y\right)=\sum_{n}\;\left[B_{n}\;\sin\left(\frac{n\pi x}{a}\right)\cosh\left(\frac{n\pi y}{a}\right)\right]\\ +T_{\text{hot}}+\left(T_{\text{cold}}-T_{\text{hot}}\right)\;\frac{x}{a}, \end{array}$$ (8)

which satisfies the boundary conditions shown in Eqs. (4–7). The boundary condition shown in Eq. (7) is used to determine the constant terms *B_n_*. For the remainder of the derivation, *T* will be used in place of *T*(*x*, *y*) for simplicity. The first and second derivatives of the solution, Eq. (8), are

$$\frac{\partial T}{\partial x}=\sum_{n}\;\left[B_{n}\;\frac{n\pi }{a}\;\cos\left(\frac{n\pi x}{a}\right)\cosh\left(\frac{n\pi y}{a}\right)\right]+\frac{\left(T_{\text{cold}}-T_{\text{hot}}\right)}{a},$$ (9)

$$\frac{\partial^{2}T}{\partial x^{2}}=\sum_{n}B_{n}\left(-{\left(\frac{n\pi }{a}\right)}^{2}\right)\sin\left(\frac{n\pi x}{a}\right)\cosh\left(\frac{n\pi y}{a}\right),$$ (10)

$$\frac{\partial T}{\partial x}=\sum_{n}B_{n}\left(\frac{n\pi }{a}\right)\sin\left(\frac{n\pi x}{a}\right)\sinh\left(\frac{n\pi y}{a}\right),$$ (11)

$$\text{and}\;\frac{\partial^{2}T}{\partial x^{2}}=\sum_{n}B_{n}{\left(\frac{n\pi }{a}\right)}^{2}\sin\left(\frac{n\pi x}{a}\right)\cosh\left(\frac{n\pi y}{a}\right).$$ (12)

Equations (10) and (12) show that the solution satisfies the Laplace equation, Eq. (3). Substitution of Eqs. (8) and (11) into Eq. (7) yields

$$\begin{array}{c} \lambda \sum_{n}\left[B_{n}\left(\frac{n\pi }{a}\right)\sin\left(\frac{n\pi x}{a}\right)\sinh\left(\frac{n\pi b}{a}\right)\right]\\ +\;h\{\sum_{n}\left[B_{n}\;\sin\left(\frac{n\pi x}{a}\right)\cosh\left(\frac{n\pi b}{a}\right)\right]+T_{\text{hot}}\\ +\;\left(T_{\text{cold}}-T_{\text{hot}}\right)\frac{x}{a}\}=hT_{\text{amb}}, \end{array}$$ (13)

which can be put into the form of a Fourier sine series:

$$\begin{array}{c} \sum_{n}B_{n}\;\sin\left(\frac{n\pi x}{a}\right)\;\left[\frac{\lambda n\pi }{a}\;\sinh\left(\frac{n\pi b}{a}\right)+h\;\cosh\left(\frac{n\pi b}{a}\right)\right]\\ =h\left[T_{\text{amb}}-T_{\text{hot}}-\left(T_{\text{cold}}-T_{\text{hot}}\right)\frac{x}{a}\right]. \end{array}$$ (14)

The term in brackets on the left side of Eq. (14) is a constant. Using the definition of the Fourier sine series [6] yields the coefficients *B_n_*:

$$\begin{array}{c} B_{n}\;\left[constant\right]=\frac{2}{a}\int_{0}^{a}h\left[T_{\text{amb}}-T_{\text{hot}}-\left(T_{\text{cold}}-T_{\text{hot}}\right)\frac{x}{a}\right]\\ \sin\left(\frac{n\pi x}{a}\right)dx. \end{array}$$ (15)

The integration yields

$$B_{n}=\frac{\frac{2}{a}\;\left[h\left(T_{\text{amb}}-T_{\text{hot}}\right)-\frac{a}{n\pi }\left(1-\cos\left(n\pi \right)\right)+h\left(T_{\text{cold}}-T_{\text{hot}}\right)\frac{a}{n\pi }\cos\left(n\pi \right)\right]}{\frac{\lambda n\pi }{a}\;\sinh\left(\frac{n\pi b}{a}\right)+h\;\cosh\left(\frac{n\pi b}{a}\right)}.$$ (16)

The resulting temperature profile can be used to determine the convective heat loss by integrating the temperature profile over the specimen surface, thereby obtaining

$$Q=\int_{0}^{a}\int_{0}^{b}h\;\left[T\left(x,y\right)-T_{\text{amb}}\right]dy\;dx,$$ (17)

and multiplying the result by 8 since the two-dimensional approximation of the three-dimensional problem involves half the height of one of the four surfaces of the specimen exposed to convection. The results show that the convective heat losses for the ceramic and metal are 0.028 W and 0.017 W, respectively, for a typical 1 W conductive heat flow. This yields a total convective heat loss of 4.5 %. This value is based on heat losses over the entire length of the specimen. The pertinent heat loss should be calculated only over the distance over which the temperature measurement is made, which is a fraction of the total specimen length. The heat loss over the distance in which the measurements are made is at most 10 % of this calculated value of 4.5 %. This short length over which measurements are typically made is another significant advantage of this measurement method.

## 4. Specimen Preparation

Due to the necessarily small size of the specimens, care must be taken in specimen preparation. All of the specimens received are in the form of disks, ranging from 3 cm to 7 cm in diameter, coated on one side. A cross-section specimen of 3.5 mm to 4 mm square was initially cut from the original disk with a low-speed diamond saw, in order to minimize damage to the coating microstructure. The cut surfaces of the specimen were then dry polished with SiC papers of decreasing particle size to remove the damaged surface material from the saw cuts. As the abrasive particle size decreases, the depth of damage decreases. Bonded-diamond platens were used for the final polishing step on the side of the specimen to be viewed by the microscope which ensured that the surface remained flat, as well as smooth. The specimen was held in a polishing rig designed to prepare specimens for viewing by transmission electron microscopy (TEM). Diamond-slurry lapping is the best form of final polish [7], but the slurry can fill pores with oils that are hard to remove. Thus the final step was to polish with bonded-diamond platens lubricating only with water. Following polishing, specimens were ultrasonically cleaned in high-purity ethanol, then air-dried to remove artificial impurities due to polishing from the existing pores of the coatings. Since the infrared microscope detector operates at wavelengths from 8 μm to 12 μm, a final polish to an average roughness of 6 μm would be theoretically sufficient to provide a surface smooth enough for diffraction-limited spatial resolution, but polishing was continued down to 0.5 μm. The final polish resulted in a scratch-free surface that was flat enough so that most of the field-of-view was in focus for a particular measurement. Only one of the four sides of the specimen needed to be polished this finely because only one side was viewed for measurement. The other three cut sides, as well as the bottom of the substrate, were polished with SiC papers down to an average roughness of 14 μm.

## 5. Results

A nominally 1 mm thick plasma-spray (PS) coating of 8 % mass fraction yttria-stabilized-zirconia was measured by both a guarded hot plate (GHP) technique and with the infrared microscopy system described here. The measurement of this coating in the GHP provides the validation of this test method. The GHP technique is an absolute, steady-state method for measurement of thermal conductivity. Numerous materials have been previously measured by this high-temperature GHP [3, 8, 9]. Thermal conductivity of PS yttria-stabilized-zirconia can be found in the literature ranging from 0.4 to 1.6 W·m^−1^·K^−1^ depending upon porosity and yttria content [1, 10, 11, 12]. A set of plasma-sprayed coatings nominally 1 mm, 2 mm, and 3 mm thick were measured in the GHP [3]. The coatings were air-plasma-sprayed at the Thermal Spray Laboratory of the State University of New York (SUNY) at Stony Brook. Each specimen had a 0.1 mm NiCrAlY bond coat between the substrate of 410 stainless steel and a coating of yttria-stabilized zirconia of mass fraction 8 % yttria.

**Fig. 5:**
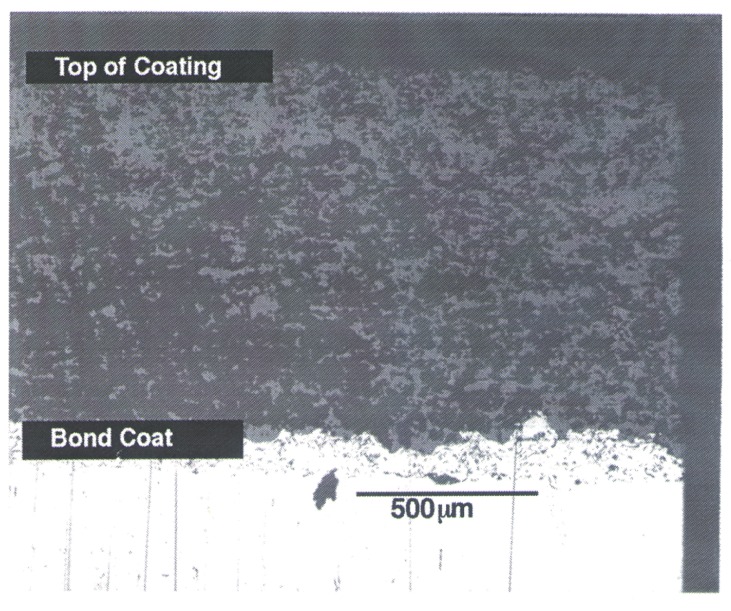
Optical micrograph of the 0.87 mm thick plasma-spray coating on a 0.1 mm thick NiCrAlY bond coat on a stainless steel substrate.

Figure 5 is an optical micrograph of the 0.87 mm (nominally 1 mm) coating measured by both the GHP technique and with the infrared microscopy system. To promote adhesion, the stainless steel substrate was sandblasted before the bond coat was applied. The application of the thin bond coat results in a very rough surface for application of the ceramic coating. The coating has a porosity of 16.5 %, measured by size and mass using weighted x-ray density values of 6.072 g·cm^−3^ for tetragonal, 6.175 g·cm^−3^ for cubic, and 5.796 g·cm^−3^ for the monoclinic phase of 8 % mass fraction yttria-stabilized zirconia. PS coatings of this composition are considered to contain only the tetragonal phase [10]. However, plasma-sprayed yttria-stabilized zirconia coatings containing all three phases of zirconia, monoclinic, tetragonal, and cubic, have been reported [13, 14]. Specimens prepared at the same laboratory using the same spray parameters and with an equivalent starting powder were analyzed using neutron diffraction [15]. By using a full Rietveld refinement analysis, the as-sprayed coating had all three phases of zirconia present, with tetragonal being the dominant phase.

**Fig. 6:**
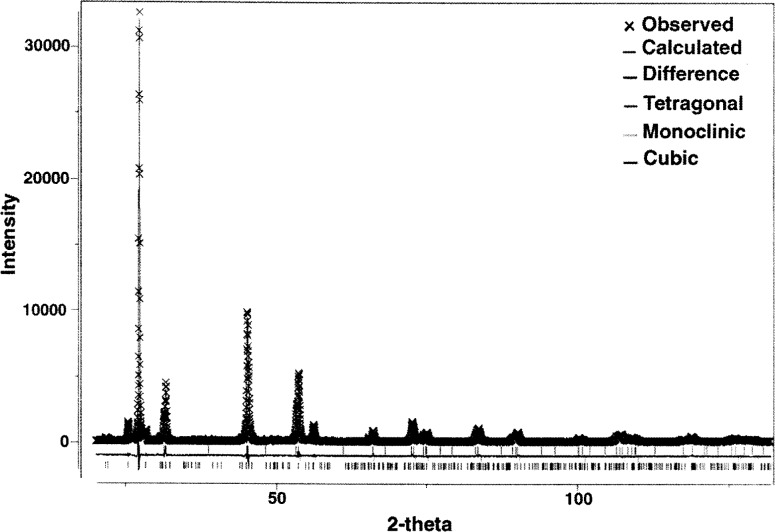
X-ray diffraction result for the 0.87 mm thick plasma-spray coating.

**Fig. 7:**
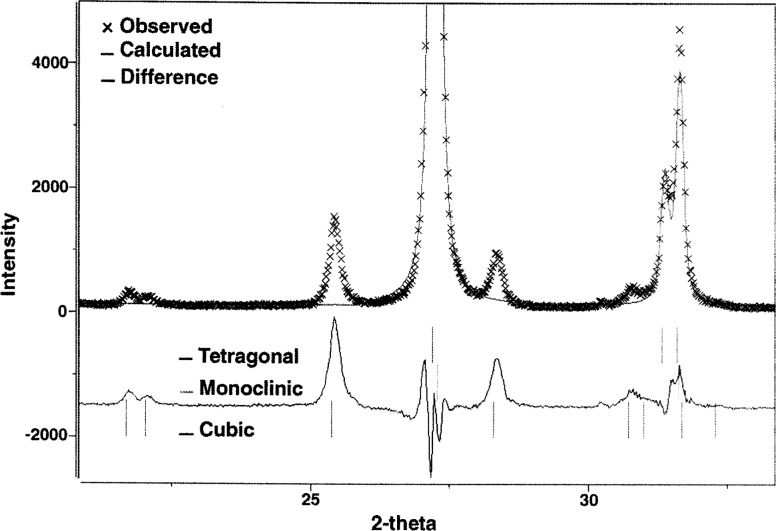
Enlarged view of part of the x-ray spectrum of the plasma-spray coating showing a calculated fit using only the tetragonal phase.

**Fig. 8:**
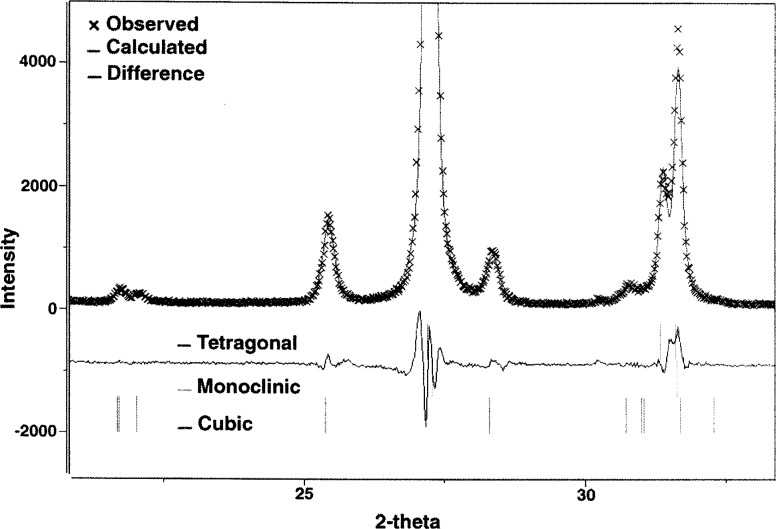
Enlarged view of part of the x-ray spectrum of the plasma-spray coating showing a calculated fit using both tetragonal and monoclinic phases

The PS coating was measured using x-ray diffraction and the phase composition determined using the Rietveld-refinement method [16]. Because of a heavy diffraction-peak overlap, it is difficult to accurately determine volume fractions and even prove the existence of all three phases of yttria-stabilized zirconia. This especially applies to the cubic and tetragonal phases, in particular when present in small quantities. To minimize this problem, the x-ray diffraction pattern was collected using CuKβ radiation, thus eliminating uncertainty and additional peak overlap introduced by the CuKα_1,2_ doublet. Figure 6 shows the diffraction pattern. An enlarged view centered around 27° is shown in Fig. 7. The line through the data is from a Rietveld refinement using only the tetragonal phase. The line below the data shows the difference between the data and the refinement result. When the monoclinic phase is included in the refinement, shown in Fig. 8, the fit to the data is very good. A small addition of cubic phase in the Rietveld refinement results in only a 0.1 % better fit. We therefore cannot state conclusively that the cubic phase exists in this specimen. The refined lattice parameters and mass fractions for all three phases that result from the best-fit refinement are given in Table 1. Mass fractions given assume no texture in all three phases. An attempt to introduce different preferred orientations failed, which indicates that the coating is texture free.

**Fig. 9:**
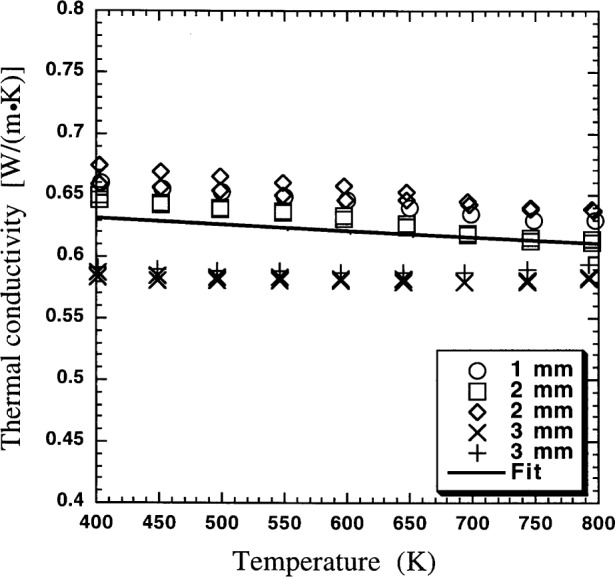
Results of thermal conductivity measurement of three thicknesses of 8 % mass fraction yttria-stabilized-zirconia coatings deposited by air-plasma spray.

**Fig. 10:**
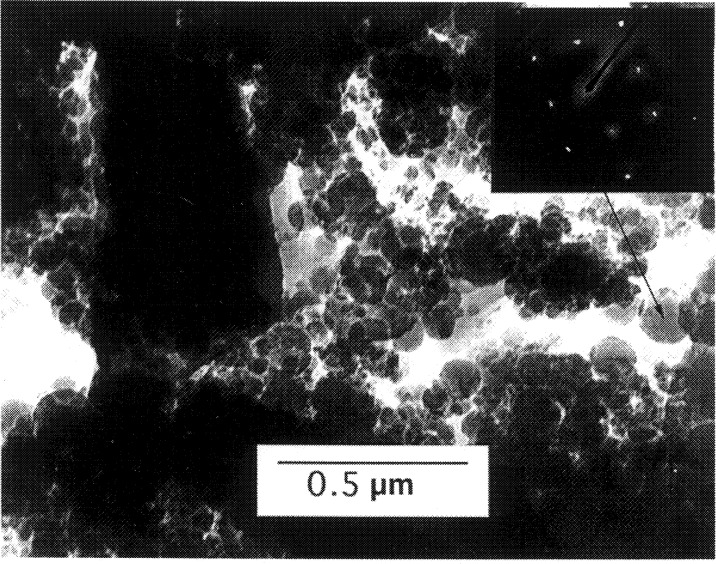
Transmission electron micrograph of the 8 % mass fraction yttria-stabilized-zirconia coating showing the small crystallite size within the splats.

Figure 9 shows the results of GHP measurements of the thermal conductivity λ of the PS coating. Even though the specimen includes a bond coat and a metallic substrate, the GHP measurements give the thermal conductivity of just the coating. Near room temperature, where the infrared microscope measurements are made, the thermal conductivity λ is 0.62 W·m^−1^·K^−1^. The thermal conductivities of these types of coatings are expected to be almost independent of temperature because of the strong phonon and geometric scattering mechanisms at work. The disordered lattice of stabilized zirconia, with numerous oxygen vacancies, scatters phonons [17]. Since the microstructure is composed of splats, there are thermally resistive interfaces between the splats. The grain structure within the splats is very fine. Figure 10 shows a transmission electron micrograph of a PS coating. The grains within the splats are on the order of 100 nm in diameter. The inset in Fig. 10 shows an electron diffraction pattern from the indicated grain. The diffraction pattern shows that the grain is either cubic or tetragonal.

**Fig. 11:**
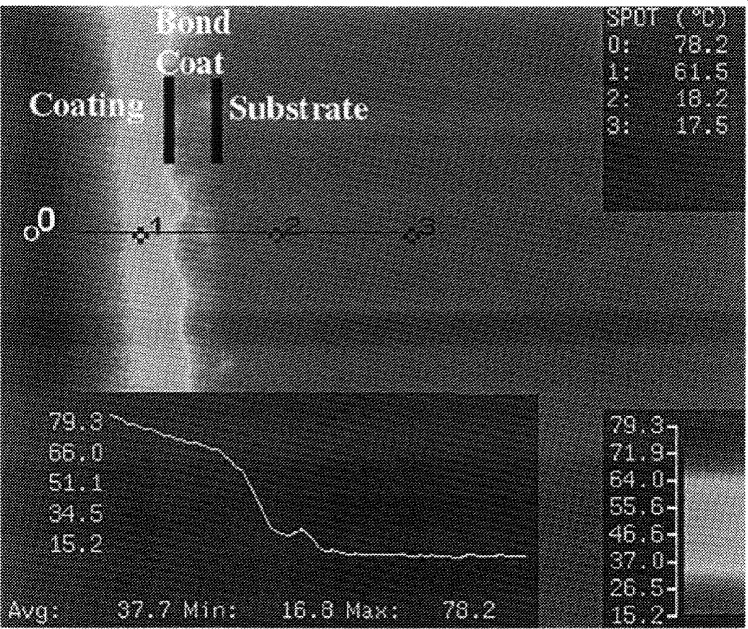
Infrared image of the 8 % mass fraction yttria-stabilized-zirconia plasma-spray coating showing the line along which the temperature profile is measured and the temperature spots are placed for thermal conductivity data acquisition [13].

Figure 11 is an infrared micrograph showing a representative measurement of thermal conductivity of the coating [18]. The designations 0 through 3 index the spots for temperature measurement. The spots are placed via software at specific locations at precise 5 μm intervals. The line shown in the figure going through the temperature spots determines the location of measurement of the temperature profile along the line. The white temperature profile is shown in the window at the lower left corner of the image, with temperatures given in degrees Celsius. Notice in the temperature-profile window that along the coating the temperature profile has a smooth slope corresponding to the thermal resistance of the coating. The sharp temperature drop corresponds to the interfacial thermal resistance at the coating/bond coat interface. The flattening slope corresponds to the thermal resistance of the NiCrAlY bond coat. The bump in the thermal profile is due to ceramic particle contamination in the bond coat. The horizontal slope is due to the substrate, which has a thermal conductivity almost 40 times higher than that of the coating. Measurements were made at 6 different specimen locations, with repeated tests at each location. The average thermal conductivity λ of the coating for the 12 measurements is 0.58 W·m^−1^·K^−1^ ±12 %, which compares well with the GHP result of 0.62 W·m^−1^·K^−1^. The agreement of these data with the GHP measurement demonstrates the validity of the experimental method. A value of 0.65 W·m^−1^·K^−1^ has been reported for a low-density yttria-stabilized-zirconia specimen of 8 % mass fraction of yttria that was air-plasma-sprayed and measured with the laser-flash technique [11, 19].

## 6. Uncertainty

This measurement method is comparative, so uncertainties from systematic effects propagate when measuring the heat flow of the known substrate material and similarly when using this measurement to determine thermal conductivity of the coating. Equation (1) is the basis for calculating both the heat-flow rate *Q* from the known thermal conductivity of the substrate material, and the subsequent thermal conductivity λ of the coating. Since all of the factors are in the form of products or quotients, if the uncertainties are expressed in relative terms, a summation in quadrature of the individual relative uncertainties gives the combined relative standard uncertainty [20]. Table 2 shows the relative standard uncertainties for the measurement of both heat flow *Q* obtained from a measurement of the substrate, and thermal conductivity λ from a measurement of the coating. The substrate and coating components have been separated for clarity.

Equation (1), applied to the substrate material, is used to measure heat flow *Q* through the specimen. From a signal-to-noise viewpoint, the temperature-difference measurement of the substrate is less certain than that for the coating due to the relatively high thermal conductivity of the substrate, and therefore low measured temperature difference. The level of uncertainty in the thermal conductivity λ of the metallic substrates is 5 %. The cross-sectional area *A* through which heat flows has a level of uncertainty of 0.5 %. The measurement of temperature difference Δ*T* is affected by thermal noise because the measurement is done in air. In addition, the scanner itself has significant internal thermal noise. According to the manufacturer, 85 % of the radiation that is measured by the detector comes from the microscope lens and other internal sources, rather than from the specimen. As mentioned earlier, a stream of temperature data for each temperature spot is averaged to minimize this effect of thermal noise. Based on repeated experiments, when the heat flow is low enough that the measured temperature difference *T* between the two temperature spots on the substrate is less than 0.3 K the measurement repeatability is greater than 25 %, and therefore unreliable. However, most measurements have a substrate temperature difference of about 1.0 K. The relative standard deviation of the substrate temperature difference data is slightly over 4 %, so the relative standard uncertainty of the temperature measurement is about 5 %. For measurements with a substrate temperature difference of less than 0.3 K, the standard deviation does not change significantly, but the thermal conductivity results show an uncertainty in reproducibility greater than 25 %, which is considered unacceptable.

Temperature spots are placed on the field-of-view via software at precise coordinates. The 1.6 mm square field is divided into a 140 × 140 grid available for spot placement. This divides the field up into 11.4 μm regions. The manufacturer gives no claim on the precision of the temperature spot placement, so linear distance testing was done in-house. Measurement of length Δ*x* by the infrared microscope was verified by measuring a 125 μm copper grid used to support specimens for transmission electron microscopy. Based on these measurements, the relative standard uncertainty in measurement of linear distance is 2 %. Since the field-of-view of the microscope is fixed and the temperature spots are placed on a grid via software, the repeatability is excellent.

As mentioned earlier, the individual relative standard uncertainties can be summed in quadrature to yield the combined relative standard uncertainty since Eq. (1) shows all factors as products or quotients [20]. Summing the relative standard uncertainties in quadrature for the measurement of heat flow yields a resulting relative combined standard uncertainty for the heat flow rate *Q* of 7.4 %. Using this result and taking into account the standard uncertainties once again for temperature difference Δ*T*, distance between temperature spots Δ*x*, and cross-sectional area *A* the combined relative standard uncertainty for measurement of thermal conductivity λ is 9.2 %. A relative expanded uncertainty of the measurement of λ is about 18 %, by using a coverage factor *k* of 2 [20].

## 7. Conclusions

A steady-state, comparative method for determining the thermal conductivity of thermal-barrier coatings has been developed. This method has been validated by measuring a PS coating that was previously measured by an accepted steady-state technique. The measurement of the PS coating using the infrared microscope yields a value for thermal conductivity of 0.58 W·m^−1^·K^−1^ 12 %. This compares well with the measured value of 0.62 W·m^−1^·K^−1^ from the GHP. The combined relative expanded uncertainty of the infrared microscope measurements is 18 % at a 95 % confidence interval. The GHP has a lower limit on coating thickness for reliable measurement of about 150 μm, whereas the thermal microscopy system can measure coatings as thin as 20 μm, in principle, which allows steady-state measurement of thermal conductivity at a size scale an order of magnitude smaller than other methods.

The use of an infrared microscope enables temperature profile measurement across a specimen. This ability will be exploited in the future in an attempt to quantify the interfacial thermal resistance between different materials.

## Figures and Tables

**Table 1:** Phase composition mass fractions *w* and lattice parameters *a*, *b*, and *c*, and the angle *β* of the three crystallographic phases in yttria-staliilized zirconia coating of mass fraction 8 % yttria, obtained by x-ray diffraction

Phase	*w* %	*a* nm	*^b^* nm	*c* nm	*β* °
Tetragonal	87.0(1)	0.36157(1)		0.51551(1)	
Monoclinic	11.6(1)	0.51660(8)	0.52022(8)	0.53221(7)	99.156(8)
Cubic	1.4(2)	0.5099(1)			

**Table 2:** Relative standard uncertainties for the measurement of thermal conductivity

	*Q* Heat flow rate, substrate %		λ Thermal conductivity, coating %
Substrate thermal conductivity λ	5	Heat flow rate *Q*	7.4
Cross-sectional area *A*	0.5	Cross-sectional area *A*	0.5
Temperature difference Δ*T*	5	Temperature difference Δ*T*	5
Measurement length Δ*x*	2	Measurement length Δ*x*	2
Total	7.4	Total	9.2

## Footnotes

1 Certain commercial equipment, instruments, or materials are identified in this paper to foster understanding. Such identification does not imply recommendation or endorsement by the National Institute of Standards and Technology, nor does it imply that the materials or equipment identified are necessarily the best available for the purpose.
